# Supplementary material for: Potential Use of Ascophyllum nodosum as a Biostimulant for Improving the Growth Performance of Vigna aconitifolia (Jacq.) Marechal
Source: Plants (Basel). 2021 Nov 2;10(11):2361. doi: 10.3390/plants10112361 (PMC8625043; doi:10.3390/plants10112361)
Supplement: Supplementary file 1 [file plants-10-02361-s001.zip › plants-1361937-supplementary.pdf]

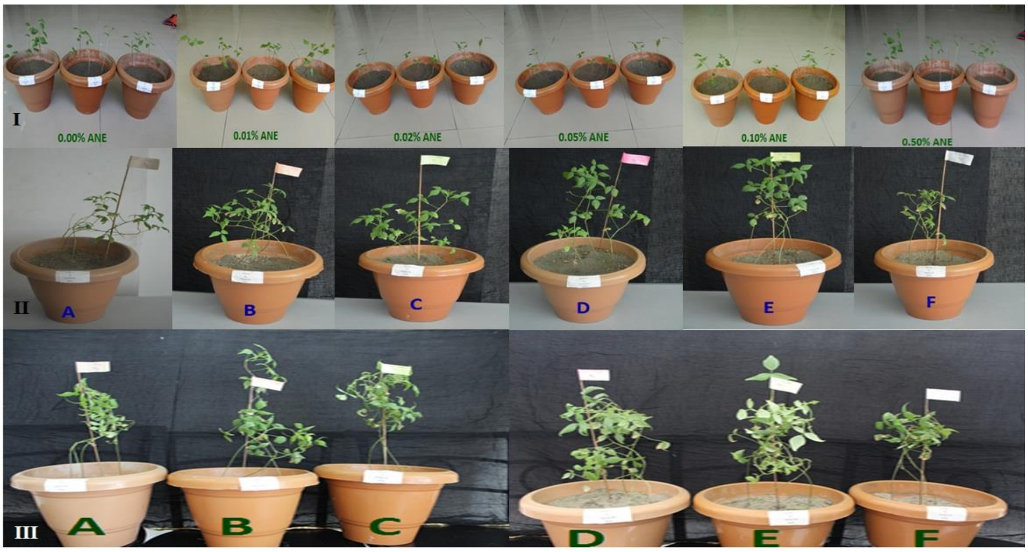

Figure S1. Shoot growth of *V. acontifolia* on (I) 15th day, (II) 30th day and (III) 75th day after Pot Foliar Application (PFA) of ANE at (A) 0.00% (B) 0.01% (C) 0.02% (D) 0.05% (E) 0.10% (F) 0.50%.

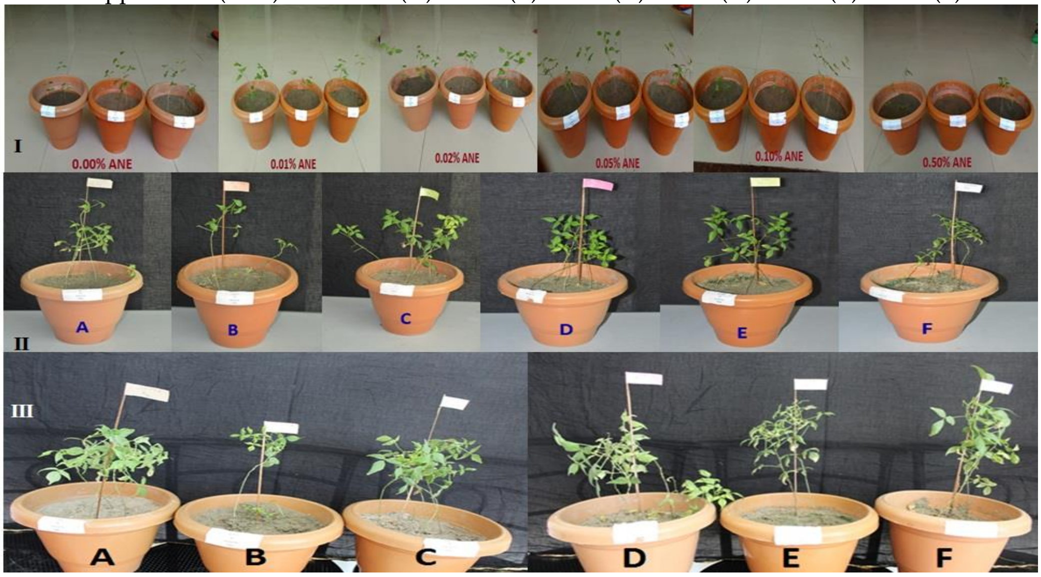

Figure S2. Shoot growth of *V. acontifolia* on (I) 15th day, (II) 30th day and (III) 75th day after pot root application (PRA) at (A) 0.00% (B) 0.01%) (C) 0.02% (E) 0.10% (F) 0.50% of ANE.
